# Facile Preparation of Lightweight Natural Rubber Nanocomposite Foams with High Wear Resistance

**DOI:** 10.3390/polym16091226

**Published:** 2024-04-27

**Authors:** Hua Jin, Fuquan Deng

**Affiliations:** 1 College of Design, Wenzhou Polytechnic, Wenzhou 325035, China; 13867132694@163.com; 2 College of Art and Design, Shaanxi University of Science and Technology, Xi’an 710021, China

**Keywords:** rubber foam, nanoclays, wear-resistant

## Abstract

The light weight and excellent mechanical properties of rubber foam means that it is widely applied in the aerospace, automobile, and military industries. However, its poor wear resistance contributes directly to a short service life and a waste of resources. Therefore, the design and development of high-wear-resistance rubber foam are of great importance. In this work, some nanoclay/rubber composite foams were prepared by blending NR/EPDM with different kinds of nanoclays containing layered double hydroxide (LDH), montmorillonite (MMT), and attapulgite (ATP) to indicate the effects of the kinds of nanoclays on the wear resistance and mechanical properties of nanoclay/rubber composite foams. The kinds of nanoclay/rubber composite foams were investigated by Fourier transform infrared spectroscopy, scanning electron microscopy, and X-ray diffraction. The results showed that nanoclay has heterogeneous nucleation in composite foamed materials. The wear resistance of the composite foam materials with added nanoclay was significantly improved, and the MMT of the lamellar structure (increased by 43.35%) and LDH (increased by 38.57%) were significantly higher than the ATP of the rod-like structure (increased by 13.04%). The improvement in the wear resistance of the matrix was even higher. Compared with other foams, the wear resistance of the OMMT–NR/EPDM foam (increased by 58.89%) with a lamellar structure had the best wear resistance. Due to the increase in the lamellar spacing of the modified OMMT, the exfoliation of worn rubber molecular chains has little effect on the adjacent molecular chains, which prevents the occurrence of crimp wear and further improves the wear resistance of composite foaming materials. Therefore, this work lays the foundation for the manufacturing of rubber foams for wear-resistant applications.

## 1. Introduction

The light weight, heat insulation, and sound insulation properties of rubber foam mean that it is widely applied in medical, transportation, aerospace, and military products [1,2,3]. However, to meet the application requirements in special environments, it is necessary to develop high-performance foam with superior physical and mechanical properties, such as flame-retardant foams, abrasion-resistant foams, and biodegradable foams [4,5,6]. Researchers have found that the poor wear resistance of foams affects the mechanical and processing costs of components from the nanoscale to the macroscale [7]. Therefore, much attention is being given to improving the wear resistance of rubber foams.

Surface engineering and matrix strengthening are considered as key strategies for the improvement of material wear resistance [8,9,10,11]. A careful modification of the structure and composition of a surface contributes to higher wear resistance of the surface layer than the substrate. On the other hand, good composition control and structure design within a bulk material contribute to high wear resistance without sacrificing the mechanical properties of the material [12]. Tong et al. [13] proposed a novel epoxy-based composite of abradable seal coating, which is a self-lubricating epoxy-based coating with a 0.26 friction coefficient and thermal properties, achieved by adding 20 vol% graphite and 50 vol% to hollow microspheres. James et al. [14] provided a method to produce water–based coatings with enhanced abrasion resistance for the first time. Hyerin et al. [15] fabricated thermoplastic elastomer nanocomposites with improved wear properties by using thermoplastic polyether elastomers (TPEEs) and surface-modified carbon black (CB), and the results showed that the wear resistance of the surface-modified CB/TPEE composite showed about four times more wear resistance. Yin et al. [16] reported a new approach to in situ synthesizing high-entropy composites (HECs) with high age-hardening ability and superior wear resistance.

A lot of articles have been published about rubber foam, but few have touched on the abrasion resistance of rubber foam. For example, researchers have successfully prepared rubber foam using natural rubber (NR) [17,18,19], ethylene propylene terpolymer (EPDM) [20,21], silicone rubber (QR) [22], acrylonitrile butadiene rubber (NBR) [23], butadiene styrene rubber (SBR) [24,25], and so on. Zhang et al. [26] studied the effect of azodicarbonamide as a foaming agent on the morphology, vulcanization kinetics, and physical properties of natural rubber foam, and the influence of nanofiller on the mechanical properties of natural rubber composites. Guerra et al. [27] prepared environmentally friendly starch bio-foam using natural rubber as a raw material. But the tearing property of natural rubber after foaming is poor when EPDM and natural rubber matrixes are selected to compound, and they do not meet the strength requirements of foaming materials in production and application. Moreover, wear resistance is also an important performance factor of rubber products, but no one has studied the wear resistance of foamed rubber, so research on the wear resistance of foamed materials based on natural rubber is a new approach.

Adding fillers is the most efficient method to improve the properties of polymer materials. Researchers use cellulose nanocrystals [28], carbon black [29], and nanosilica [30] as reinforcements to improve the mechanical properties of materials. Jiang et al. [31] reported that a small amount of nanoclay can significantly enhance the physical, mechanical, and thermal properties of polymers, but a large amount of nanoclay would agglomerate in the polymer matrix, resulting in poor polymer properties. He et al. [32] indicated that modified organic nanoclay could provide better dispersion, faster curing speeds, and a higher curing density. But up to now, no one has studied the effect of nanoclays with different morphologies and structures on the properties of foam materials based on natural rubber.

In this study, natural rubber and EPDM were selected as a foaming matrix. In order to investigate the effect of nanoclays with different or similar morphologies on the properties of NR-based foaming materials, we added attapulgite, hydrotalcite, and nano–montmorillonite into the rubber matrix to prepare nanocomposites. In addition, nano–montmorillonite was modified to prepare modified montmorillonite/natural–rubber–based foam materials. The mechanical properties, thermal mechanical properties, wear resistance, and microstructure of the composites are discussed.

## 2. Materials and Methods

### 2.1. Materials

Natural rubber (3L, Vietnam) was purchased from Shanghai East tiger Industrial Co., Ltd. (Shanghai, China). EPDM (3720P, ethylene content 69%, third elastomer content (ENB) 0.5%, Mooney viscosity 20) was supplied by the Dow Chemical Company (Midland, MI, USA). Sulfur (S) and dicumyl peroxide (DCP), used as curing agents, were provided by Tianjin Fuchen Chemical Reagents Factory (Tianjin, China). The curing auxiliary diphenylpropylthiazole disulfide (M) was supplied by Hebi Lian Hao chemical Limited by Share Ltd. (Hebi, China). Azodicarbonamide (AC–3000H), used as a foaming agent (decomposition temperature range was 160–180 °C), was purchased from Dongguan Haili Holding Group Co., Ltd. (Dongguan, China). Zinc oxide (ZnO) and stearic acid (St), used as activators, were purchased from Dongtai Hongyuan Chemical Factory (Dongtai, China) and Tianjin Tianli Chemical Reagent Co., Ltd. (Tianjin, China), respectively. White carbon black (WCB) and solid paraffin (Sp), used as reinforce agents, were purchased from TaiChang resin materials Co., Ltd. (Dongguan, China) and Petro China Co., Ltd. (Qingyang, China), respectively. Sodium montmorillonite (Na-MMT), attapulgite (ATP), and hydrotalcite (LDH) nanoclays were provided by Zhangjiakou Qinghe chemical plant (Zhangjiakou, China).

### 2.2. Organic Modification of Nano-Montmorillonite

An amount of 15 g Nano–montmorillonite was placed in a 500 mL flask; ethanol and water were added in a 9:1 ratio to the flask as a solvent, and 0.2 mol/L hydrochloric acid solution was used to adjust the pH between 3 and 4. Then, a silane coupling agent (KH–570) was added with mass fraction of 5% and the mixture was heated to 80 °C in an oil bath with mechanical stirring and reflux condensation for 8 h. After the reaction was finished, the solution was cooled to room temperature naturally and then centrifugally cleaned with deionized water and anhydrous ethanol (4000 rap 6 min). Centrifugation was adopted to neutralize and remove the silane coupling agents which were not involved in the reaction. Wet MMT clay was collected, cut it into small particles, and put on a watch glass. It was dried at 80 °C in an oven for 24 h. The dry clay was ground into a powder, collected, and marked as OMMT.

### 2.3. Preparation of Natural-Rubber-Based Foams

The natural rubber and EPDM were masticated in an internal mixer (S(X) M-0.5L-K (Suyan, Wuxi, China, 3 L)) for 10 min at 100 °C. Then, the reinforcing agent, activator, blowing agent, nanoclay, crosslinking agent, and curing auxiliaries were added into the internal mixer. Subsequently, the compound was placed on an open two-roll mill (XH-401C, Xihua, Dongguan, China) to mix at 70 °C. It was ensured that the filler mixture of the rubber matrix was uniform. The compound was stored at room temperature for 24 h to release the remaining stress in the mixing process. Ultimately, the foam was prepared using compression molding at 170 °C under a pressure of 10 MPa by a flat-panel curing press (XH-406, Xihua, Dongguan, China). The ingredients in the rubber compounds were (in phr) as follows: natural rubber, 70; EPDM, 30; WCB, 12; Sp, 5; ZnO, 6; St, 3; DCP, 0.6; S, 0.4; M, 0.167; AC-3000H, 4. The content of nanoclay in the different compounds is shown in Table 1.

### 2.4. Characterization

#### 2.4.1. Fourier Infrared Test

The Fourier Infrared Spectrometer VERTE 70 from Bruker, Ettlingen, Germany, was adopted for the test. After the solid sample was fully dried, it was mixed with KBr particles and ground into a compact powder with a wave number range of 500 cm^−1^ to 4000 cm^−1^ and a resolution of 1 cm^−1^.

#### 2.4.2. X-ray Diffraction (XRD)

The NR/EPDM foams were cut into 10 × 10 × 5 mm cubes. XRD patterns were recorded in the range of 2θ = 10–60° by 4-step scanning with a D8 Advance (Bruker, Ettlingen, Germany).

#### 2.4.3. Vulcanization Characteristics

According to the national standard of vulcanization characteristics [33], to determine the vulcanization characteristics, a rotorless vulcanizer—MM4130C2—was used to test the vulcanization performance of the sample.

#### 2.4.4. Physical and Mechanical Properties

The shrinkage was calculated by the following equation: S (%) = (Lm ± Lc)/Lm × 100% (1), where S is the shrinkage of the foam, Lm is the length of the original foam, and Lc is the length of the final foam after cooling. The mechanical properties were conducted using a Desktop Tensile Strength Tester AI-3000 (Gotech, Taiwan, China) with a loading rate of 500 mm/min under a 500 N load cell. The Young’s modulus of all samples was determined by the slope of the linear region of the stress–strain curve. The toughness was calculated by the area under the stress–strain curve. The compressive stress–strain curve of the foam was measured by a Servo Control Universal Testing Machine (AI–7000–LA, Gotech, Taiwan, China) with a loading rate of 5 mm/min under a 3000 N load cell. The sample diameter was 50 mm. The compression shear hysteresis curves of FOAM and F-OMMT were tested by a dynamic testing machine (Instron 8803, Shanghai, China).

#### 2.4.5. Differential Scanning Calorimeter

Under the condition of a nitrogen atmosphere (flow rate of 40 mL/min), the heating rate was 10 °C/min and the temperature range was −80–200 °C.

#### 2.4.6. Thermogravimetric Analyzer

We used the thermogravimetric analysis (,TA instrument Q500, Newcastle, DE, USA). We used about 5 mg of the sample at a heating rate of 10 °C/min and a temperature range of 25–600 °C. The nitrogen flow rate was 60 mL/min, and the weight of the residue was used to calculate the carbon content in the sample.

#### 2.4.7. Akron Abrasion Resistance Test

The Akron abrasion tester was used to test the abrasion resistance of the foamed material samples. A certain quantity of rubber compound was vulcanized and foamed in a circular mold. After the foam material was formed, it was placed on an Akron abrasion tester with a load of 500 g. After pre-grinding for 15 min, we removed the wear debris from the sample, weighed the sample mass, called it the initial mass, and recorded it as *m*_0_. Then, we started the wear test. When the wear distance reached 1.61 Km, we removed the sample, removed the wear debris, and weighed the final mass, which was recorded as *m*_1_. We calculated the abrasion volume according to the following formula:

Δ*V* = (*m*_0_ − *m*_1_)/ρ (1)

ρ—sample density; unit: grams/cubic centimeter (g/cm^3^).
*m*_0_—initial mass; unit: grams (g).
*m*_1_—final mass; unit: grams (g).
∆*V*—abrasion volume; unit: cubic centimeter/1.61 Km (cm^3^/1.61 Km).

## 3. Results and Discussion

**Figure 1:**
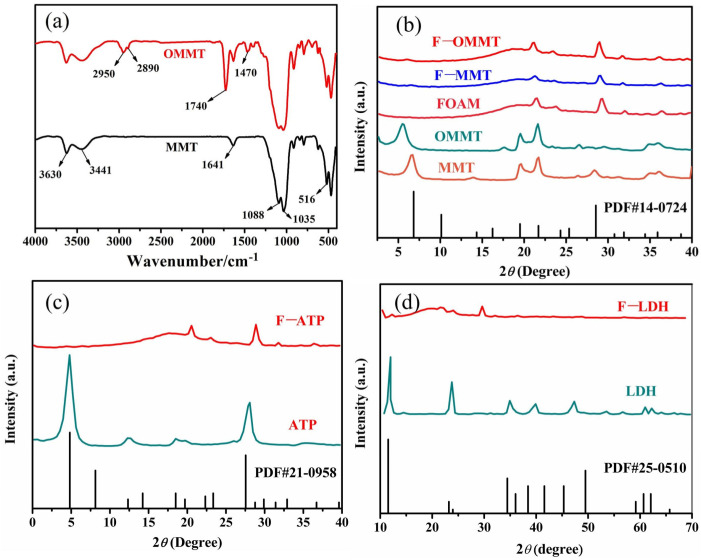
(**a**) Infrared spectra of nano-montmorillonite and (**b**–**d**) X-ray diffraction patterns of NR/EPDM composite foams with nanoclay.

Figure 1a shows the FTIR spectra of the pre-modification and post-modification of nano–montmorillonite. The stretching vibration peak of Al-OH, belonging to nano-montmorillonite at 3630 cm^−1^, can be seen from the infrared spectrum. At 3441 cm^−1^ and 1641 cm^−1^, the absorption peaks caused by the -OH bending and stretching vibrations in the water molecules of the nano-montmorillonite lamellae are observed. The characteristic peaks at 1088 cm^−1^ and 1035 cm^−1^ belong, respectively, to Si-O-Si inside and outside the stretching vibration peak. The symmetrical bending vibration absorption peak of Si-O is marked at 516 cm^−1^ [34,35]. It can be seen from the picture that the corresponding characteristic peaks of the nano-montmorillonite did not disappear after treatment with KH-570, indicating that the layered structure of Na-MMT remained intact. Moreover, there are two new absorption peaks at 2950 cm^−1^ and 2890 cm^−1^ which belong to the stretching vibrations of methyl and methylene as a result of KH-570 [36], and the peaks of the carbonyl group of KH-570 appear at 1740 cm^−1^. The peak at 1470 cm^−1^ is the bending vibration absorption peak of the C-H bond. This proves that Na-MMT was successfully modified by KH-570.

Figure 1b shows the XRD pattern of the MMT particles and modified MMT particles and the related nanoclay/NR-based foam materials. The characteristic peaks of the nano-montmorillonite treated by the silane coupling agent (2θ_OMMT_ = 5.94°, d_OMMT_ = 1.49) can be seen to move to a lower angle in Figure 1b. According to Bragg’s law [37] (2dsinθ = nλ), we can see that the interlayer spacing of the nano-montmorillonite increased by 0.23 after the silane coupling agent KH-570 was modified. It is proven that the modification of montmorillonite with the silane coupling agent has a better effect on increasing interlayer spacing. As we can see from Figure 1c, there was no peak (2θ_ATP_ = 7.4°) in the XRD pattern in the F-ATP foam nanocomposite. It is generally accepted that the attapulgite (ATP) was completely exfoliated in the foamed materials [38]. Figure 1b,d show that the peaks of montmorillonite (MMT, 2θ_MMT_ = 7°, d_MMT_ = 1.26) and hydrotalcite (LDH, 2θ_LDH_ = 11.7°) appear clearly in the foamed materials, respectively, indicating that nano-hydrotalcite and nano-montmorillonite exist in the composite foamed materials as exfoliated or intercalated structures.

**Figure 2:**
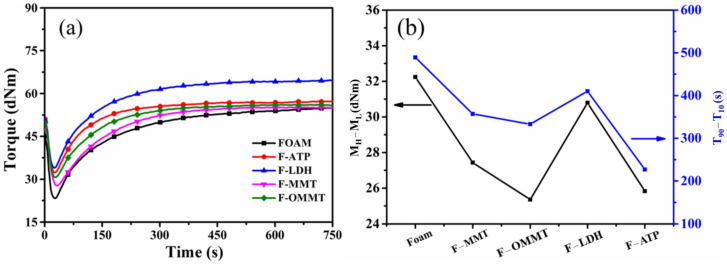
The curing behavior of NR/EPDM composite foams with nanoclay: (**a**) curing curve; (**b**) M_H_–M_L_ and T_90_–T_10_.

Figure 2 exhibits the effects of various types of nanoclay on the curing behaviors of the NR-based foam materials derived from the moving die rheometer measurements at 170 °C. The curing vulcanization parameters, such as initial torque (M_i_), highest torque (M_H_), lowest torque (M_L_), delta torque (∆M), scorch time (t_10_), optimum curing time (t_90_), and curing rate index (CRI = 100/(t_90_–t_10_)) are given in Table 2. Figure 2a shows the curing curve of the NR/EPDM composite foams with nanoclay; from the point of curing time, the nanoclay fillers not only reduced the scorch time, but also decreased the optimum time needed for the vulcanization molding of composites. Figure 2b shows the M_H_–M_L_ and T_90_–T_10_ of the NR/EPDM composite foams with nanoclay. The size of the M_H_–M_L_ value reflects the crosslink density of the composite: the higher the M_H_–M_L_ value, the higher the crosslink density. The size of the T_90_–T_10_ value represents the vulcanization rate of the composite: the smaller the value of T_90_–T_10_, the faster the vulcanization rate. As can be seen from Figure 2b, the size of the M_H_–M_L_ value decreases after the nanoclay is added, so the crosslink density of the system decreases. The size of the T_90_–T_10_ value decreases after the nanoclay is added, so the vulcanization rate of the system increases. This is because of the role of nanoclay as a heterogeneous nucleation site in the foamed composite, which makes it easier to generate bubbles in the composite. As shown in Table 2, the initial torque of the composite foam was always increased, independent of the type of nanoclay. Because the nanoclay increased the viscosity of the system and also created physical bonding with the polymer, which decreased the chain mobility, we can observe an increase in initial torque.

So, we can obtain the following conclusion: nanoclay not only plays a role in increasing viscosity and hindering the movement of polymer chains in polymer systems, but also catalyzes the vulcanization of polymers. Moreover, the curing rate index shows the speed of the composite vulcanization process intuitively [39].

**Figure 3:**
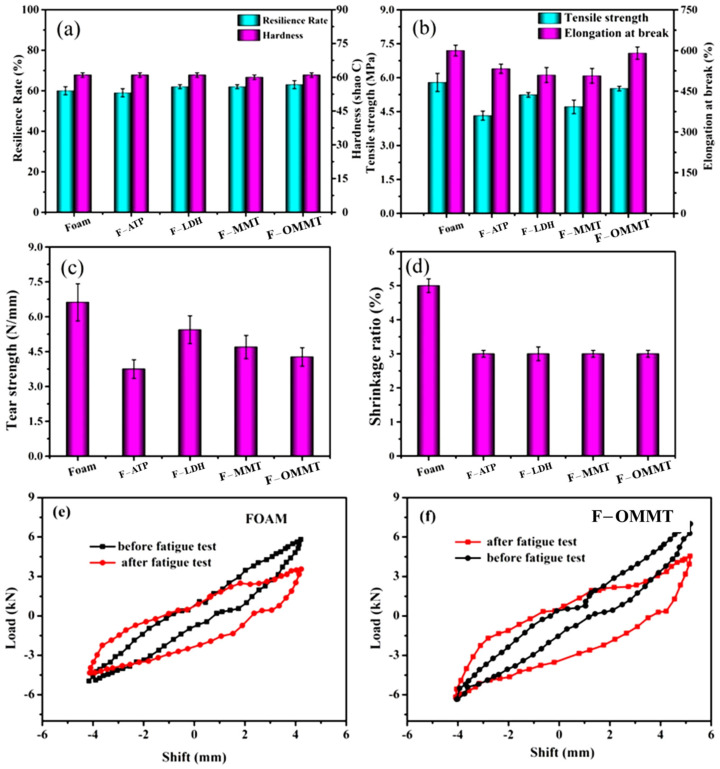
Mechanical properties of the nanoclay/natural-rubber-based foamed materials: (**a**) resilience rate and hardness, (**b**) tensile strength and elongation at break, (**c**) tear strength, (**d**) shrinkage rate, and the compression shear hysteresis curves of (**e**) FOAM and (**f**) F–OMMT.

Figure 3 shows the mechanical properties of the nanoclay/natural-rubber-based foam materials, including hardness, resilience rate, tensile strength, elongation at break, shrinkage, and tear strength. It can be seen from Figure 3a that nanoclay has little effect on the hardness and resilience of foamed materials, whether directly filled or modified by organic materials. This is because the hardness and resilience of polymer materials are mainly determined by the strength of the polymer matrix material itself, the crosslinking density of the polymer, and the degree of foaming. Nanoclay has almost no effect on this property. It can be seen from Figure 3b,c that the tensile and tear strength of the polymer materials decreased with the addition of nanoclay. As mentioned above, this is due to the poor compatibility of nanoclay in the polymer matrix. Nanoclay not only adsorbs the blowing agent in the polymer matrix, forming a heterogeneous nucleation point in the foam, but creates defective points in the matrix phase interface, so the performance of the polymer materials deteriorates. The strength of the composites synthesized by the treated montmorillonite increased slightly because of the better compatibility between the nano-montmorillonite and the polymer after the organic treatment, which meant that the nano-montmorillonite played the role of polymer reinforcer. As for the change in elongation at break, it has been reported [40] that elongation at break decreases with an increase in filler content in polymer systems.

The effect of the nanoclay on the shrinkage of the polymer materials is revealed in Figure 3d. It is obvious that adding nanoclay into the polymer reduced the foamed materials’ shrinkage rates. The nanoclay used in this paper can reduce the shrinkage of polymers by 2%, which may be due to the crystalline structure of the nanoclay in the polymer collective. The rigid group acts as a framework support in the heat dissipation and shrinkage process of the composite foam after vulcanization, which hinders the movement of the polymer chain and reduces the shrinkage of the polymer material. As shown in Figure 3e–f, the maximum load of FOAM and F-OMMT was reduced from 5.7 kN and 7.1 kN to 3.4 kN and 4.5 kN after the fatigue test, respectively. The compression shear hysteresis curves after fatigue were fuller than before fatigue. This is because the introduction of OMMT improved the cellular structure of F-OMMT. 

**Figure 4:**
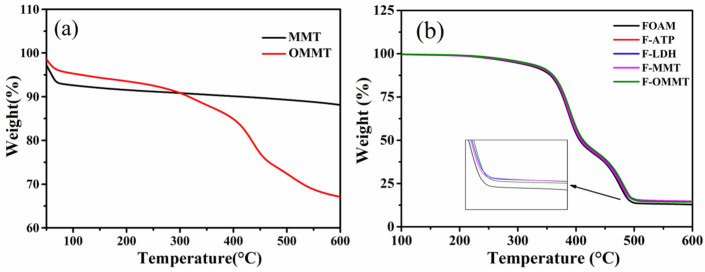
Thermal decomposition curves: (**a**) MMT and OMMT; (**b**) nanoclay/NR/EPDM composite foams.

Figure 4 shows the thermal decomposition curves of the nanoclay/natural rubber composite foam materials and nano-montmorillonite before and after the modification. It can be seen from Figure 4a that the weight loss at 75 °C in the nano-montmorillonite is caused by the desorption of water molecules adsorbed on the surface of the nano-montmorillonite. A long plateau area appears at 75–550 °C with little mass loss, and the slow decomposition after 550 °C is attributed to the gradual desorption of water adsorbed by the nano-montmorillonite layers. At 600 °C, the residual rate of the sample is 88%. The nano-montmorillonite modified by the silane coupling agent KH-570 has a similar decomposition tendency before 300 °C. The thermal weight loss at 300–600 °C is due to the thermal degradation of the organic components in the modified nano-montmorillonite. The maximum decomposition rate appears at 438 °C and the decomposition rate is 0.195. The final residual rate of the sample is 67%, which is lower than the unmodified nano-montmorillonite because of the degradation of organic components in the sample. The thermal decomposition curves of the nanoclay/natural rubber composite foam materials can be seen in Figure 4b. The thermal weight loss at 200–500 °C is caused by the thermal degradation behavior of the rubber-based foam materials. The initial thermal degradation (TiD), T_5_, and T_30_ are listed in Table 3. The maximum decomposition rates at 386 °C and 473 °C are attributed to the rapid decomposition of NR and EPDM at corresponding temperatures. Finally, the residual rate of F–MMT > F–OMMT > FOAM is similar to the thermal decomposition curve of montmorillonite. F–MMT > F–OMMT is due to the fact that the modified nanofiller contains a certain amount of organic components in the same mass fraction, which are gradually destroyed and degraded during the heating process. The residual ratio of the foamed composite filled with nanoclay is higher than that of the unfilled foamed composite because nanoclay cannot be completely decomposed at 600 °C. Thus, it can be seen that the introduction of nano-montmorillonite has no obvious effect on the thermal decomposition trend of natural-rubber-based foam materials.

**Figure 5:**
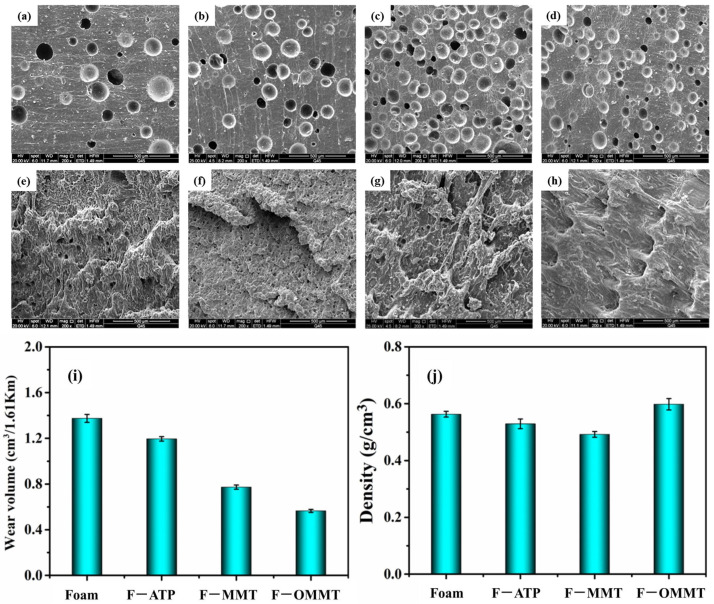
Morphology of nanoclay/NR-based foams before and after wear: (**a**,**e**) foam, (**b**,**f**) F–ATP, (**c**,**g**) F–MMT, (**d**,**h**) F–OMMT. (**i**,**j**) Abrasion resistance and density of the nanoclay/natural-rubber-based foams.

Figure 5 shows the wear volume of the nanoclay/natural-rubber-based composite foams and the scanning electron microscope photos of the composite foams before and after the Akron abrasion test. Wear mass is not strictly discussed because of the different density of the foamed materials. Therefore, this test mainly focuses on the wear volume of the foamed materials.

As can be seen from Figure 5i, the abrasion resistance of the composite foam material is obviously improved after filling with nanoclay. Among them, nano-montmorillonite with a lamellar structure (improved by 43.45%) has a more obvious improvement than attapulgite with a rod-like structure (improved by 13.04%). The nano-montmorillonite modified by the silane coupling agent KH-570 as a filler improves the abrasion resistance of the composites most excellently (improved by 58.89%). It is possible that the untreated nanoclay has worse compatibility in the composites, provides more heterogeneous nucleation sites, forms more dense bubbles after initial wear, exposing nanoparticles, and forms abrasive debris of different sizes, causing aggravation in the process of continuous wear [41]. The effect of the nanoclay on the density of the composite materials is shown in Figure 5j. It can be seen from the picture that the density of the composites decreased with the addition of ATP and MMT. This is because they act as heterogeneous nucleating agents in the matrix, providing heterogeneous nucleating sites, adsorbing blowing agents, and making cell growth easier. As a result, material density decreased. The density of the MMT composites prepared by the silane coupling agent (KH-570) increased because the treated MMT had good compatibility in the composites, thus playing the role of a polymer reinforcement filler and increasing the density of the materials.

It can be seen from Figure 5a that the natural rubber foams without nanoclay had an uneven size and a small number of bubbles. Figure 5b–d show that the size of the foam cell becomes uniform and smaller, and the number of foam cells becomes larger when the nanoclay particles are filled. This is due to the introduction of nanoclay and more heterogeneous nucleation sites being formed, providing more foaming sites for the blowing agent. During the vulcanization process, the aggregation of the foaming agent was avoided and the formation of macropores was prevented. In Figure 5d, the number of bubbles is smaller than the unmodified nano-montmorillonite, proving that the compatibility is better than the unmodified nanoclay.

Figure 5e–h reveal the scanning electron microscopy (SEM) results of the nanoclay composite foams after the Akron wear test. Figure 5e shows an abrasion photograph of the natural rubber foam without added nanoclay. It can be seen that there is no obvious abrasion morphology. During the grinding process, the rubber structure sheds quickly. Figure 5f–h show abrasion photographs of natural rubber foam prepared by adding different kinds of nanoclay. It can be seen that after filling with nanoclay, the wear morphology displays a little crimp wear. It is assumed that the crystalline structure of the nanoparticles hinders the wear behavior and thus reduces the wear of the rubber matrix during the wear process. Moreover, when the lamellar nanoclay acts as filler, a ridge-like structure appears in the wear morphology, while only curling wear occurs in the composites made of bar-like attapulgite clay. Considering that lamellar nanoclay plays a role in reducing curling wear in the composites, we modified the layered nano-montmorillonite with a silane coupling agent and studied its wear morphology. Figure 5h clearly shows that there is a regular ridge structure [42] without curl wear, which decreases the tendency of adhesion friction. We suggest that the spalling of rubber molecular chains has little effect on the nearby molecular chains due to the obstruction of uniformly dispersed modified montmorillonite lamellae in the process of Akron wear. This reduces the occurrence of crimp wear and further reduces the wear volume of the material.

**Figure 6:**
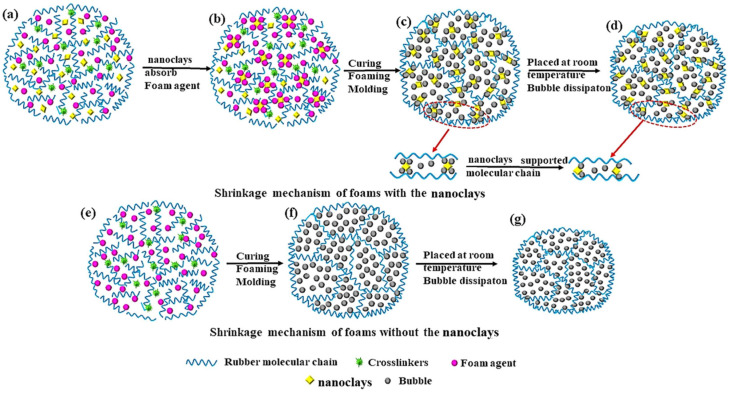
Shrinkage mechanism of the nanoclay/NR-based foams: (**a**–**d**) Anti-shrinkage process of nanoclay to NR-based foams; (**e**–**g**) shrinkage process of NR-based foams.

Figure 6 shows the shrinkage mechanism of the nanoclay/NR-based foaming materials. Figure 6a–c reveal the molecular chains supporting the nanoclay in the polymer foaming process, and Figure 6e–f show the vulcanization process of the rubber-based foaming materials without nanoclay. Figure 6d,g show the shrinkage of the foams with or without nanoclay support after gas evaporation, respectively. As we can see, in the mixing process, the nanoclay adsorbs the foaming agent and provides heterogeneous nucleation sites for the foaming agent. During vulcanization, the foaming agent is heated and foams around the nanoclay. When the gas in the polymer unit spills over the polymer shrinks, while the nanoclay acts as a molecular chain skeleton between the molecular chains. This can provide some support in the process of molecular chain shrinkage, thus effectively improving the shrinkage phenomenon of foaming materials.

**Figure 7:**
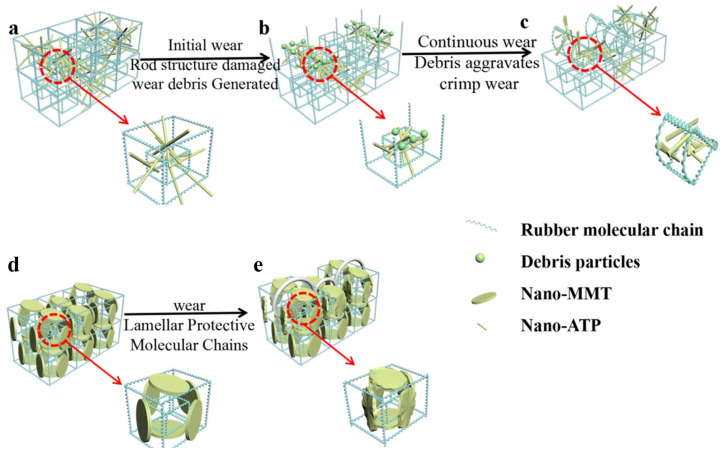
Wear mechanism of the nanoclay/NR-based foams: (**a**–**c**) the wear mechanism of tubular nano-attapulgite; (**d**,**e**) the wear mechanism of lamellar nano-montmorillonite.

Figure 7 shows the wear mechanism of the nanoclay/NR-based foaming materials: the red line indicates the wear process and the red-dotted line indicates the local enlargement. It is believed that the introduction of lamellar nano-montmorillonite and tubular nano-attapulgite improves the wear resistance of natural-rubber-based composite foams, which is due to the fact that the rigid structure of nanoclay can play a certain role in the wear process compared with polymer structures. It can be seen from Figure 7d,e that nano-montmorillonite intercalation between polymer layers can play a certain role in hindering wear behavior, reducing the impact of adjacent molecular chains or layers in the wear process and avoiding the occurrence of crimp wear. Figure 7e shows that the presence of nano-montmorillonite has a protective effect on the wear behavior of molecular chains. As seen in the SEM photographs, the nano-montmorillonite forms a ridge-like structure.

However, nano-attapulgite plays a minor role in the wear resistance of polymer materials. The reason is that polymer molecular chains are entangled in rod-like attapulgite clay, and rod-like attapulgite clay cannot effectively prevent the wear of other molecular chains in the wear process. We can see from Figure 7a that the rigid structure of nano-attapulgite can prevent wear at the early stage of wear. However, as the wear behavior proceeds, the tubular structure of nano-attapulgite is gradually destroyed and debris is formed on the surface of the polymer, which can be seen in the local magnification in Figure 7b. In the continuous wear process, the presence of debris will further aggravate the wear and make the polymer surface curl, as can be seen in Figure 7c.

This reveals the reason why lamellar nano-montmorillonite is superior to tubular nano-attapulgite in improving the wear resistance of polymers. In addition, lamellar nano-montmorillonite modified by a silane coupling agent not only enlarges the interlayer spacing, but also results in better dispersion and compatibility in the polymer, which improves the wear resistance of the polymer more clearly.

## 4. Conclusions

Filling with nanoclay can improve the foam structure of rubber foams and support the molecular chain role in the rubber matrix, which can effectively reduce the shrinkage of foamed materials. In addition, the influence of nanoclay on the strength of polymer materials mainly depends on the compatibility between the nanoclay and the polymer matrix. More importantly, nanoclay filling can effectively improve the wear resistance of NR-based nanocomposite foaming materials. The lamellar structure of nano-montmorillonite improves the wear resistance of the system more markedly than attapulgite with a rod-like structure. The further improvement of wear resistance also depends on the dispersion and compatibility of the nanoclay and macromolecule materials. Using nano-montmorillonite modified by a silane coupling agent (KH-570) as a filler, the wear resistance of nanocomposite foam materials based on natural rubber can be increased by 58.89%. However, the introduction of nanoclay did not play a significant role in the thermal degradation of natural-rubber-based foaming materials, and the increase in the residual amount was mainly due to the presence of nanoclay which could not be completely degraded in the polymer. The rubber-based foaming material prepared in this work can be used in motion soles in order to reduce the weight of shoes while ensuring durable performance.

## Figures and Tables

**Table 1:** The content (in phr) of nanoclay in all samples.

Sample	FOAM	F–ATP	F–LDH	F–MMT	F–OMMT
ATP	0	3	0	0	0
LDH	0	0	3	0	0
MMT	0	0	0	3	0
OMMT	0	0	0	0	3

**Table 2:** Curing characteristics of the nanoclay/natural-rubber-based foamed materials.

Sample	M_i_ (dNm)	M_L_ (dNm)	M_H_ (dNm)	∆M (dNm)	t_10_ (s)	t_90_ (s)	CRI
FOAM	45.05	23.31	55.55	32.24	28	517	0.2045
F–MMT	47.73	27.73	55.16	27.43	28	385	0.2833
F–OMMT	49.63	30.64	56	25.36	27	360	0.3003
F–LDH	50.62	33.97	64.76	30.79	25	435	0.2439
F–ATP	51	32.33	58.16	25.83	26	253	0.4405

**Table 3:** Thermal stability data of the nanoclay/NR/EPDM composite foams.

Samples	Temperature/°C			Residual Rate/%
	TiD	T_5_	T_30_	
FOAM	243.5	286.2	382.7	17.3
F–ATP	248.3	305.5	385.0	19
F–LDH	250.4	308.2	384.6	19
F–MMT	253.6	318.7	385.2	19
F–OMMT	260.4	321.4	388.1	18

T_5_ and T_30_ are the decomposing temperatures at 5% and 30% weight loss, respectively.

## Data Availability

Data are contained within the article.
